# Maternal Exposure to Microplastics and High-Fructose Diet Induces Offspring Hypertension via Disruption of H_2_S Signaling, Gut Microbiota, and Metabolic Networks

**DOI:** 10.3390/antiox15020179

**Published:** 2026-01-30

**Authors:** Chien-Ning Hsu, Chih-Yao Hou, Yu-Wei Chen, Guo-Ping Chang-Chien, Shu-Fen Lin, You-Lin Tain

**Affiliations:** 1 Department of Pharmacy, Kaohsiung Municipal Ta-Tung Hospital, Kaohsiung 801, Taiwan; cnhsu@cgmh.org.tw; 2 Department of Pharmacy, Kaohsiung Chang Gung Memorial Hospital, Kaohsiung 833, Taiwan; 3 School of Pharmacy, Kaohsiung Medical University, Kaohsiung 807, Taiwan; 4 Department of Seafood Science, National Kaohsiung University of Science and Technology, Kaohsiung 811, Taiwan; chihyaohou@nkust.edu.tw; 5 Department of Food Science and Biotechnology, National Chung Hsing University, Taichung 402, Taiwan; d112043001@mail.nchu.edu.tw; 6 Department of Pediatrics, Kaohsiung Chang Gung Memorial Hospital, Kaohsiung 833, Taiwan; 7 Center for Environmental Toxin and Emerging-Contaminant Research, Cheng Shiu University, Kaohsiung 833, Taiwan; guoping@csu.edu.tw (G.-P.C.-C.); 6101@gcloud.csu.edu.tw (S.-F.L.); 8 Super Micro Mass Research and Technology Center, Cheng Shiu University, Kaohsiung 833, Taiwan; 9 Institute of Environmental Toxin and Emerging-Contaminant, Cheng Shiu University, Kaohsiung 833, Taiwan; 10 Institute for Translational Research in Biomedicine, Kaohsiung Chang Gung Memorial Hospital, Kaohsiung 833, Taiwan; 11 College of Medicine, Chang Gung University, Taoyuan 333, Taiwan; 12 Doctoral Program of Clinical and Experimental Medicine, National Sun Yat-Sen University, Kaohsiung 804, Taiwan

**Keywords:** hydrogen sulfide, hypertension, gut microbiota, microplastics, fructose, Developmental Origins of Health and Disease (DOHaD), untargeted metabolomics

## Abstract

Maternal consumption of a high-fructose (HF) diet or exposure to microplastics (MPs) can each independently affect kidney development and increase the risk of hypertension in adult offspring, yet their combined impact remains poorly understood. Dysregulation of hydrogen sulfide (H_2_S) signaling and alterations in gut microbiota are potential mediators of this programming. Pregnant rats received either standard chow or a 60% HF diet, with half of each group additionally exposed to sulfate-modified MPs (1 mg/L) with a 5 μm diameter throughout pregnancy and lactation. Male offspring were divided into four groups (n = 7–8 per group): control, HF, MP, and HF+MP. Maternal HF or MP exposure raised offspring blood pressure (BP), with additive effects when combined, and MP exposure caused renal injury. MP treatment also suppressed renal H_2_S-generating enzymes and reduced H_2_S production. Both HF and MP exposures altered gut microbial composition linked to BP regulation and induced metabolic changes in taurine/hypotaurine and sulfur pathways, suggesting impaired H_2_S production. These results indicate that maternal HF and MP exposures interfere with H_2_S signaling, gut microbiota, and metabolic programming, highlighting the H_2_S signaling as a potential target to reduce long-term kidney and cardiometabolic risks.

## 1. Introduction

The production of plastic has surged significantly in recent decades, leading to widespread environmental contamination [1]. These plastics fragment into micro- and nanoplastics (MPs/NPs), which have been detected in diverse environmental matrices, including the food chain, raising significant public health concerns [1,2]. Simultaneously, the increased incorporation of high-fructose corn syrup (HFCS) in processed foods and drinks has substantially elevated dietary fructose consumption, raising alarms about potential health risks [3]. Many HFCS-containing drinks, such as sodas and fruit juices, are packaged in plastic, which can release hundreds to thousands of MPs per liter [4], highlighting a critical intersection between dietary habits and environmental exposures [5].

The Developmental Origins of Health and Disease (DOHaD) hypothesis posits that adverse in utero conditions may lead to both structural and functional changes during fetal development, increasing the likelihood of chronic diseases in later life [6]. Maternal fructose intake during pregnancy and lactation induces kidney programming, leading to structural and functional renal alterations that heighten offspring susceptibility to adult hypertension [7]. Similarly, maternal MP exposure leads to MP accumulation in the placenta and fetal kidneys [8,9], with emerging evidence linking such exposures to elevated risk of hypertension and kidney disease in offspring [10]. Co-exposure to MP and high fructose (HF) during critical developmental windows may therefore synergistically disrupt fetal kidney development and program offspring toward early-onset hypertension.

Hydrogen sulfide (H_2_S) signaling has emerged as a key mechanistic pathway linking environmental and dietary exposures to disease [11]. Endogenous H_2_S is primarily generated by three enzymes—cystathionine γ-lyase (CSE), cystathionine β-synthase (CBS), and 3-mercaptopyruvate sulfurtransferase (3MST)—and can also be produced via non-enzymatic reactions and gut microbial metabolism. H_2_S has a key role in kidney development, vascular regulation, and blood pressure (BP) control [12,13]. Dysregulated H_2_S signaling and gut microbiota alterations are implicated in kidney programming and hypertension [14,15]. Yet, it remains unclear whether maternal HF diets or MP exposure contribute to offspring hypertension through these mechanisms.

To address this knowledge gap, we established a two-hit maternal rat model integrating HF diet and MP exposure to investigate their combined effects on offspring hypertension and underlying mechanisms. Understanding these interactions could inform preventive strategies targeting H_2_S signaling, gut microbiota modulation, and metabolite regulation, ultimately reducing hypertension risk across generations.

## 2. Materials and Methods

### 2.1. Animal Design

All experimental procedures were approved by our institution’s Institutional Animal Care and Use Committee (IACUC), under approval number 2024061201. These activities were conducted in a facility that holds accreditation from AAALAC International. Virgin female Sprague–Dawley (SD) rats were procured from BioLASCO Taiwan Co., Ltd. (New Taipei City, Taiwan). For breeding, each female was housed overnight with a male, and mating success was verified the next morning by the observation of a vaginal plug.

Throughout pregnancy and lactation, the pregnant SD rats were provided with either a standard chow diet (n = 6) or chow containing 60% fructose (HF; n = 6). The control pregnant rats received a standard maintenance chow (Altromin 1320, Altromin Spezialfutter GmbH & Co., Lage, Germany), a cereal-based diet providing approximately 24% of energy from protein, 65% from carbohydrates, and 11% from fat, with carbohydrates derived primarily from complex polysaccharides. In contrast, the HF-exposed pregnant rats were fed a purified 60% fructose diet (Teklad TD.89247, Envigo, Madison, WI, USA), in which fructose constituted 600 g/kg of the diet and served as the principal carbohydrate source, accounting for approximately 66.8% of total caloric intake, with 20.2% from protein and 13.0% from fat. Within each dietary group, half of the pregnant mother rats were additionally exposed to 1 mg/L MPs during the same period. The polystyrene MPs (5.0 μm, green fluorescence; Lot PSG5638) were sulfate-modified, negatively charged particles custom synthesized by Magsphere Inc. (Pasadena, CA, USA), as described in our previous report [10]. MPs were supplied as a 10% solid suspension in 50 mL solution, with particle size variation within ±10%. Before dilution into drinking water, the suspension was homogenized using a vortex mixer to ensure even dispersion. Water bottles were routinely washed, and freshly prepared MP solutions were replenished biweekly to maintain exposure consistency.

The chosen dosages for the HF diet and MP exposure were informed by earlier studies conducted on rodents [10,16,17]. The analysis concentrated exclusively on male offspring, as they exhibit a heightened susceptibility to early-onset hypertension compared to their female counterparts [18]. The offspring were divided into four experimental groups (n = 7–8 per group): control (CN), maternal HF diet, maternal MP exposure (MP), and combined high-fructose plus MP exposure (HFMP).

Systolic BP measurements were obtained every four weeks using the CODA noninvasive tail-cuff system (Kent Scientific, Torrington, CT, USA). At 12 weeks of age, all offspring were humanely sacrificed. Prior to euthanasia, fecal samples were collected in the morning by gently elevating the tail to stimulate defecation, then immediately frozen at −80 °C. Anesthesia was induced with an intraperitoneal injection of combined xylazine (10 mg/kg) with ketamine (50 mg/kg). Euthanasia was then performed by administering an intraperitoneal overdose of pentobarbital (150 mg/kg). Following confirmation of death, blood samples were drawn, and kidneys were excised after systemic perfusion with phosphate-buffered saline (PBS).

### 2.2. Histology and Morphometric Study

Kidney sections (4 μm, formalin-fixed, paraffin-embedded) were stained with hematoxylin and eosin (H&E). Renal injury was blindly evaluated by scoring glomerular and tubulointerstitial damage. Up to 100 glomeruli were graded on a 0–4 scale. Tubulointerstitial injury was scored as 0–5 based on tubular degeneration, atrophy, dilation, sloughing, basement membrane thickening, and interstitial widening (0 = none; 1 < 10%; 2 = 10–25%; 3 = 25–50%; 4 = 50–75%; 5 = 75–100% involvement).

### 2.3. Renal Gene Expression Analysis by qPCR

Total RNA was extracted from kidney cortex samples, and cDNA was generated using MMLV Reverse Transcriptase kit (Invitrogen, Carlsbad, CA, USA). Quantitative real-time PCR was then carried out using the QuantiTect SYBR Green PCR kit (Qiagen, Valencia, CA, USA) on an iCycler iQ system (Bio-Rad, Hercules, CA, USA) to assess the expression of H_2_S-producing enzymes. All reactions were run in duplicate, with 18S rRNA (R18S) as the internal control. Rat-specific primers were as follows: CBS (NM_012522.2) FW 5′-ATGCTGCAGAAAGGCTTCAT-3′, RV 5′-GTGGAAACCAGTCGGTGTCT-3′; CSE (NM_017074.2) FW 5′-CGCACAAATTGTCCACAAAC-3′, RV 5′-GCTCTGTCCTTCTCAGGCAC-3′; 3MST (NM_138843.2) FW 5′-GGCTCAGTAAACATCCCATTC-3′, RV 5′-TGTCCTTCACAGGGTCTTCC-3′; and R18S (X01117) FW 5′-GCCGCGGTAATTCCAGCTCCA-3′, RV 5′-CCCGCCCGCTCCCAAGATC-3′. Relative expression was calculated using the comparative threshold cycle (2^−ΔΔCT^) method.

### 2.4. Renal H_2_S Synthesis

Renal H_2_S-producing activity was assessed as previously described [19]. Briefly, kidney tissues (1:10, *w*/*v*) were homogenized in ice-cold 100 mM potassium phosphate buffer (pH 7.4). Aliquots (430 μL) of homogenate were incubated with L-cysteine (10 mM, 20 μL) and pyridoxal 5′-phosphate (2 mM, 20 μL) at 37 °C for 30 min in sealed vials. The reaction was terminated by adding 10% trichloroacetic acid (250 μL) and 1% zinc acetate (250 μL). Following color development with ^N,N^-dimethyl-p-phenylenediamine sulfate (20 mM, 20 μL) and FeCl_3_ (30 mM, 20 μL) in HCl, absorbance was measured at 670 nm. H_2_S concentrations were quantified using NaHS standards (3.125–250 μM) and expressed as μM/min.

### 2.5. Gut Microbiome Metagenomic Analysis

Fecal microbial DNA was extracted and subjected to full-length 16S rRNA gene sequencing using the PacBio platform (Menlo Park, CA, USA) with barcoded primers for SMRTbell library construction. Sequencing was conducted in collaboration with Biotools Co., Ltd. (New Taipei City, Taiwan). Amplicon sequence variants (ASVs) were inferred and used to generate phylogenetic trees with the FastTree algorithm implemented in QIIME2. Taxonomic classification was assigned against the Greengenes reference database. Sequencing data underwent stringent quality control, including removal of low-quality reads and exclusion of chimeric sequences.

Within-sample (α-diversity) metrics, including Pielou’s evenness, the Shannon index, and Simpson’s diversity index, were calculated to describe microbial richness and distribution. Between-sample (β-diversity) differences were evaluated using partial least squares discriminant analysis (PLS-DA) in conjunction with analysis of similarities (ANOSIM). To identify discriminative taxa, linear discriminant analysis effect size (LEfSe) was applied.

Functional inference of the microbial community was performed using PICRUSt2 (v2.2.0-b) [20] based on ASVs, following standard protocols (https://github.com/picrust/picrust2/wiki, accessed on 31 December 2025). ASVs were phylogenetically placed to generate a reference–environmental tree, which was then used to predict gene family copy numbers based on enzyme classification (EC) annotations. PICRUSt2 outputs included EC gene family predictions and MetaCyc pathway abundances [21], which were visualized and analyzed using STAMP v2.1.3 [22]. Statistical comparisons between groups were performed with Welch’s *t*-test, and all results were corrected for multiple testing (Bonferroni q < 0.05).

### 2.6. Untargeted Metabolomics Analysis

Rat serum was extracted with 800 μL ice-cold MeOH containing internal standard. Supernatants were dried under N_2_, reconstituted in 200 μL MeOH, filtered (0.22 μm), and stored at −20 °C. Analyses were performed on a ThermoFisher UltiMate 3000 UHPLC coupled with a Q Exactive Orbitrap (Thermo Fisher Scientific, San Jose, CA, USA) using an HSST3 C18 column (2.1 × 100 mm, 1.8 μm). The mobile phases were 0.1% formate in water (A) and ACN (B) at 0.3 mL/min; injection volume was 5 μL, with reservoir at 4 °C and oven at 40 °C, in both positive and negative ESI modes. Raw data were processed with Compound Discoverer 3.3, including peak extraction, alignment (5 ppm tolerance, max 0.2 min shift), metabolite identification, QC correlation, gap status, and background subtraction. Peaks ≥1,000,000 intensity with ≥7 scans were retained. Identification was based on the LWHK database or online libraries (mzCloud, Metabolika, ChemSpider), considering mass error < 5 ppm, isotope similarity, and fragmentation spectra when available. QC criteria were CV < 30%, with 1 QC per 10 injections. Metabolites present in ≥25% of samples and with sample/blank ratio ≥ 3 were included. Processed tables were further analyzed with LWHK software, which assigned confidence levels (1–4): Level 1, match to LWHK standards (mass, fragmentation, and retention time); Level 2, match to mzCloud (mass and fragmentation); Level 3, accurate mass only; Level 4, unknown peaks.

### 2.7. Statistical Analysis

Data are expressed as mean ± standard error of the mean (SEM). The normality of the datasets was evaluated using the Shapiro–Wilk test to determine the appropriate statistical approach. For data meeting normality assumptions, differences among groups were assessed by one-way ANOVA with Tukey’s post hoc test; the non-normally distributed data were analyzed using the Kruskal–Wallis test followed by Dunn’s post hoc comparisons. Statistical significance was set at *p* < 0.05. All analyses were performed with SPSS version 17.0 (SPSS Inc., Chicago, IL, USA).

## 3. Results

### 3.1. Offspring Outcomes

In this study, we demonstrate that maternal HF diet and MP exposure synergistically program adverse outcomes in offspring. Mortality was absent in all groups, and body weight (BW), kidney weight (KW), and KW/BW ratio were unchanged (Table 1). However, systolic BP began to rise by week 8 in the HF and MP groups, reaching ~9 mmHg and ~13 mmHg above controls, respectively, at week 12 (Table 1). Notably, combined HF and MP exposure produced a pronounced synergistic effect, elevating BP by ~20 mmHg in the HFMP group at 12 weeks, highlighting the additive cardiovascular risk of these maternal exposures.

**Figure 1:**
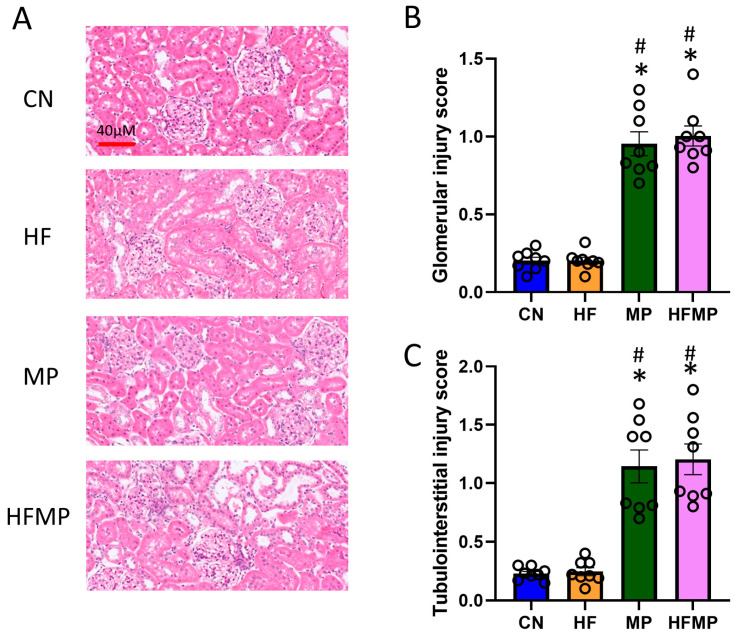
Maternal high-fructose (HF) diet and microplastic (MP) exposure impair offspring kidney morphology. (**A**) Representative hematoxylin and eosin-stained kidney sections from offspring at 12 weeks (original magnification ×20). (**B**) Glomerular injury and (**C**) tubulointerstitial injury scores are summarized in bar graphs. Data are expressed as mean ± SEM. * *p* < 0.05 vs. CN; # *p* < 0.05 vs. HF. n = 7–8 per group.

The histological analysis revealed marked glomerular and tubulointerstitial injury in MP and HFMP offspring (Figure 1A–C), indicating that MP exposure, both alone and in combination with HF, is associated with kidney injury. These findings are consistent with previous reports that maternal HF diet or MP exposure induces kidney programming and predisposes to hypertension and kidney disease [6,9].

### 3.2. H_2_S Pathway

**Figure 2:**
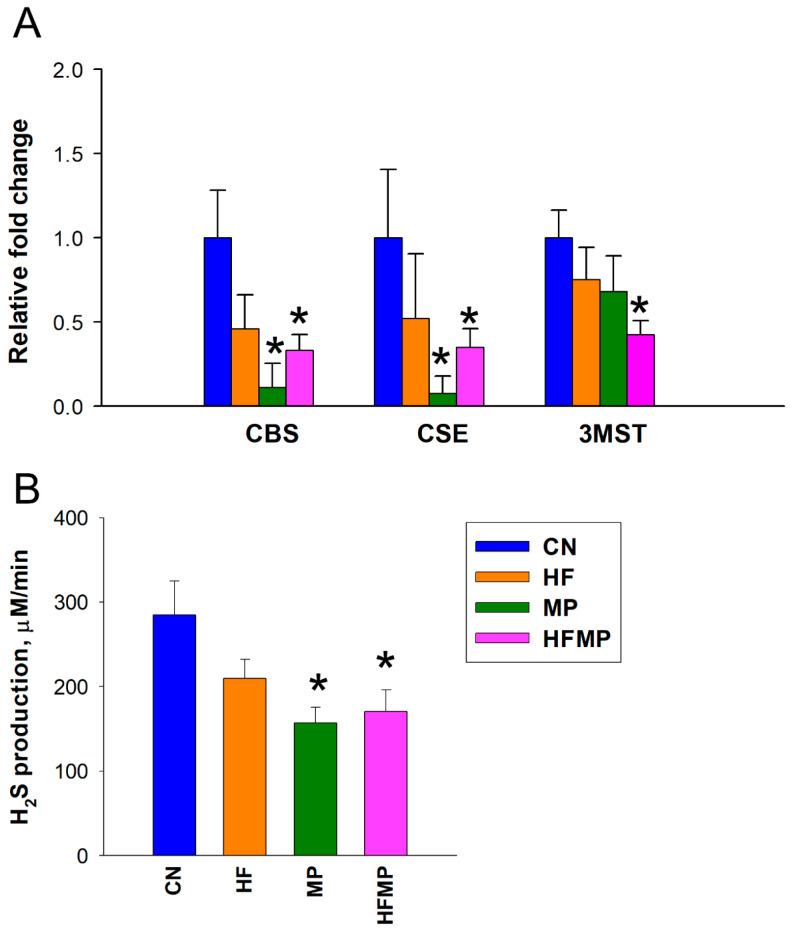
Effects of maternal high-fructose (HF) diet and microplastic (MP) exposure on the renal H_2_S pathway. (**A**) Renal mRNA expression of H_2_S-generating enzymes CBS, CSE, and 3MST in adult offspring. (**B**) Renal H_2_S production activity in vitro. Data are presented as mean ± SEM (n = 7–8 per group). * *p* < 0.05 vs. CN.

To assess the impact of maternal HF diet and MP exposure on the H_2_S pathway, we measured renal mRNA expression of H_2_S-producing enzymes and in vitro H_2_S production in offspring kidneys. As shown in Figure 2A,B, maternal HF diet alone did not alter the expression of H_2_S-generating enzymes or H_2_S production. In contrast, MP exposure significantly reduced renal mRNA levels of CBS and CSE compared with controls (CBS: CN 1.00 ± 0.28 vs. MP 0.11 ± 0.14 fold change, *p* < 0.05; CSE: CN 1.00 ± 0.40 vs. MP 0.07 ± 0.10 fold change, *p* < 0.05). Combined HF and MP exposure similarly decreased CBS and CSE expression (CBS: CN 1.00 ± 0.28 vs. HFMP 0.33 ± 0.09 fold change, *p* < 0.05; CSE: CN 1.00 ± 0.40 vs. HFMP 0.35 ± 0.11 fold change, *p* < 0.05) and H_2_S production (CN 284.9 ± 39.9 vs. HFMP 170.6 ± 25.5 μM/min, *p* < 0.05) and additionally reduced 3MST expression (CN 1.00 ± 0.17 vs. HFMP 0.42 ± 0.08 fold change, *p* < 0.05) in offspring kidneys, indicating a more pronounced disruption of the H_2_S pathway. Renal expression and catalytic activity of the H_2_S-generating enzymes were significantly altered by maternal MP exposure, whereas the HFMP group exhibited a pattern comparable to MP alone, indicating exposure-specific modulation of the H_2_S system rather than a causal relationship with BP.

### 3.3. Gut Microbiota Composition

**Figure 3:**
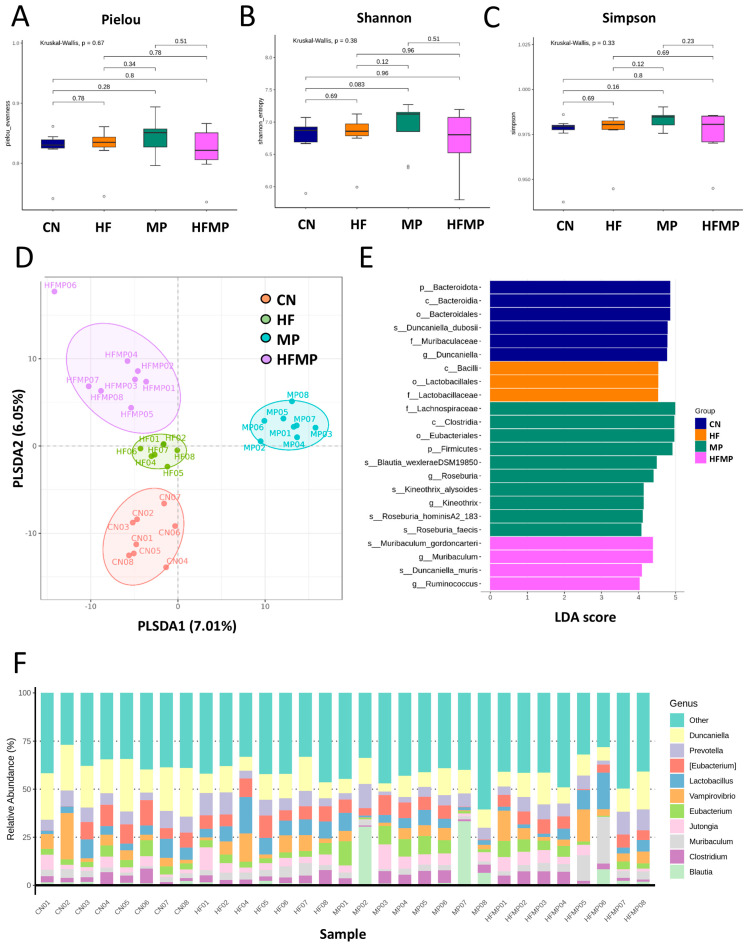
Effects of maternal high-fructose (HF) diet and microplastic (MP) exposure on offspring gut microbiota. (**A**) Pielou evenness index. (**B**) Shannon diversity index. (**C**) Simpson diversity index. (**D**) Partial least squares discriminant analysis (PLS-DA) of bacterial taxa across the four groups. (**E**) Differential bacterial taxa identified by linear discriminant analysis effect size (LEfSe) with an LDA score > 4. (**F**) Relative abundance of the top 10 gut microbial genera. n = 7–8 per group.

To determine whether maternal HF diet or MP exposure alters the offspring gut microbiota, we performed 16S rRNA gene sequencing on fecal samples collected from 12-week-old rats. Measures of α-diversity—including the Pielou evenness index (Figure 3A), Shannon diversity index (Figure 3B), and Simpson diversity index (Figure 3C)—showed no significant differences in species richness or evenness among the four groups. In contrast, PLS-DA revealed distinct clustering of gut microbial communities (Figure 3D), which was further supported by ANOSIM, indicating significant differences between groups (all *p* < 0.05).

Linear discriminant analysis effect size (LEfSe) identified taxa with differential abundance among groups (Figure 3E). The control (CN) group exhibited enrichment of *Duncaniella dubosii* and its corresponding higher taxonomic levels (genus, family, order, class, and phylum). The MP group showed increased relative abundance of genera *Roseburia* and *Kineothrix*, while the HFMP group was enriched in *Muribaculum* and *Ruminococcus*. As shown in Figure 3F, the predominant genera across all groups included *Duncaniella*, *Prevotella*, *Eubacterium*, *Lactobacillus*, *Vampirovibrio*, *Muribaculum*, *Clostridium*, *Blautia*, and *Jutongia.*

**Figure 4:**
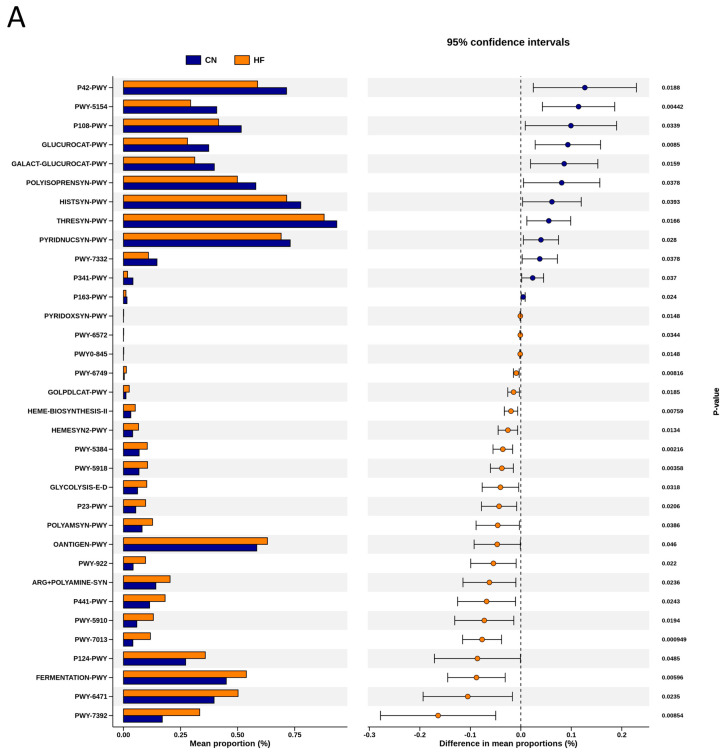

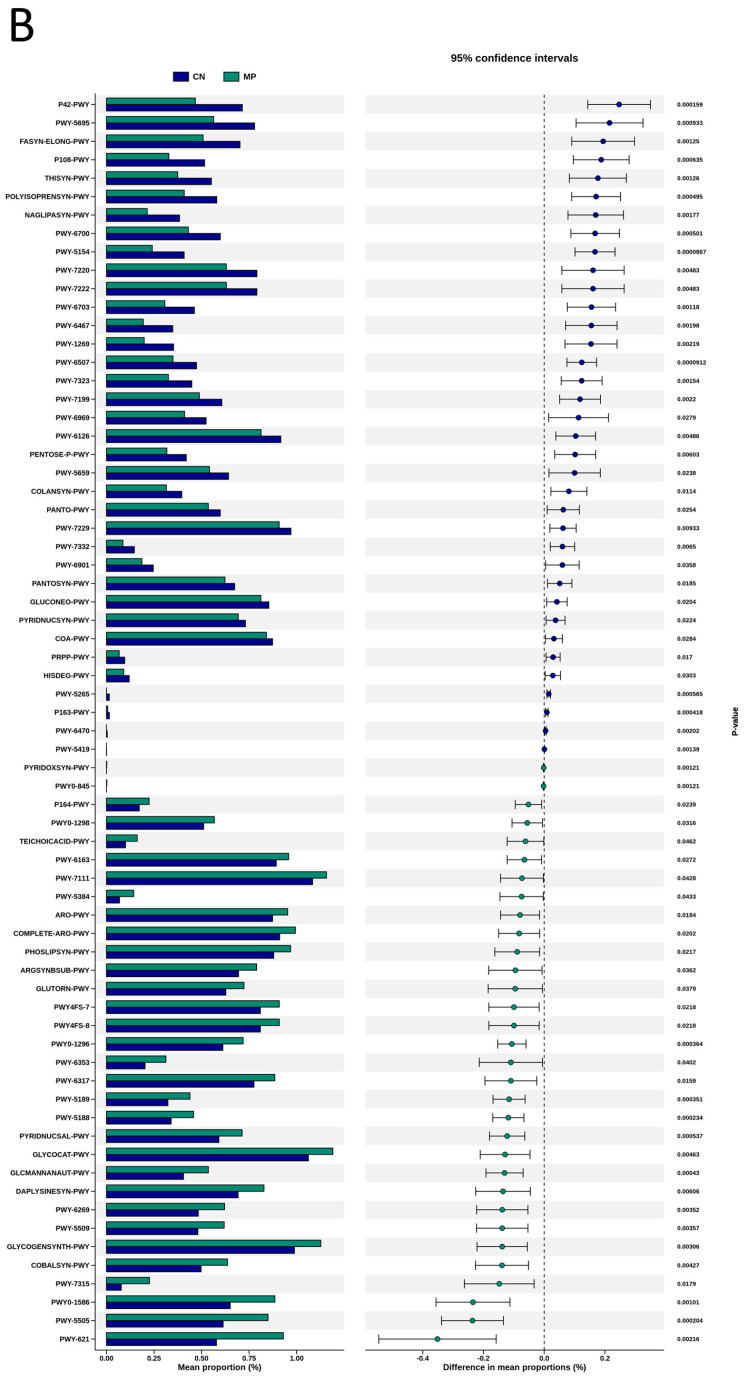

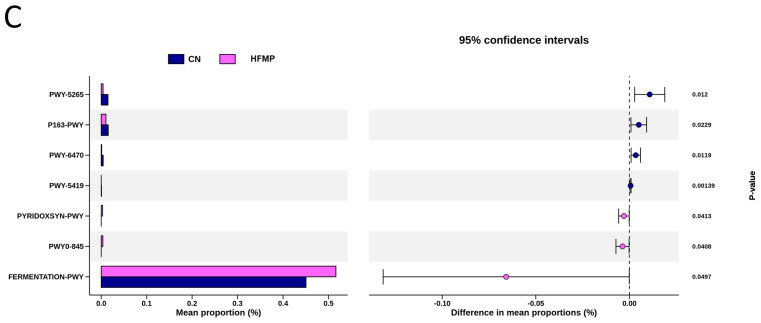
Several functionally relevant enzyme classifications, found using the MetaCyc database, were significantly altered by (**A**) high-fructose (HF) diet, (**B**) microplastic (MP) exposure, or (**C**) their combined exposure. n = 7–8 per group.

To explore the functional capacity of the gut microbiota, a PICRUSt2-based analysis of MetaCyc pathways was performed. Compared with the CN group, 34, 68, and 7 MetaCyc pathways were significantly altered in the HF (Figure 4A), MP (Figure 4B), and HFMP (Figure 4C) groups, respectively. Notably, pathway PWY-7323 (superpathway of sulfate assimilation and cysteine biosynthesis), which encompasses assimilatory sulfate reduction to sulfide and cysteine biosynthetic steps, was significantly suppressed by MP exposure compared to the CN group (*p* = 0.00154) (Figure 4B).

**Figure 5:**
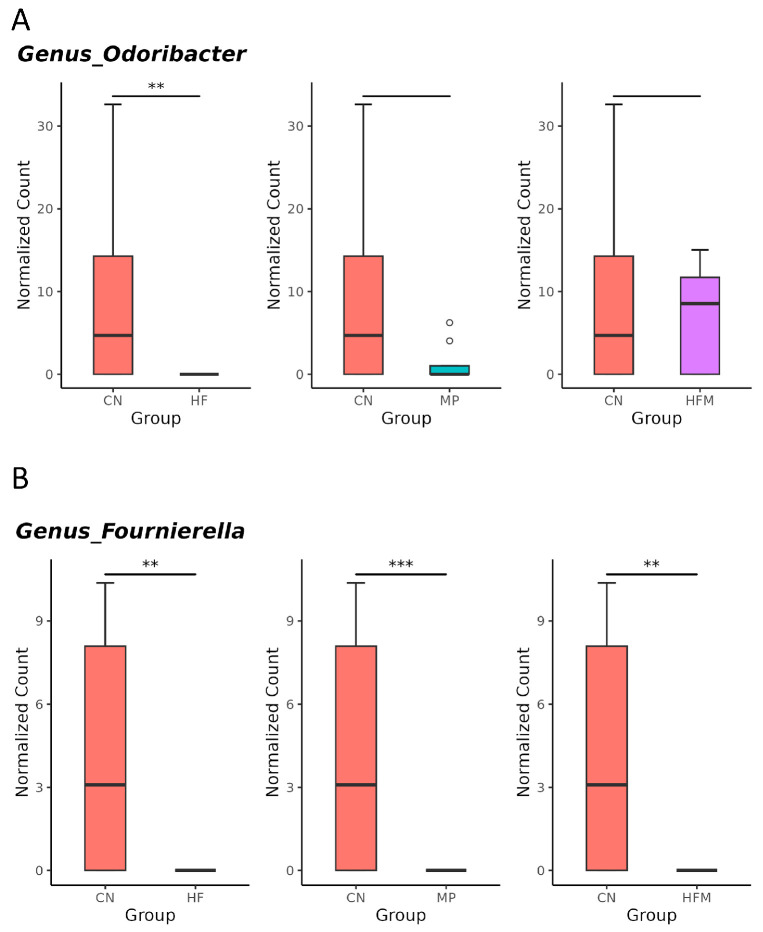

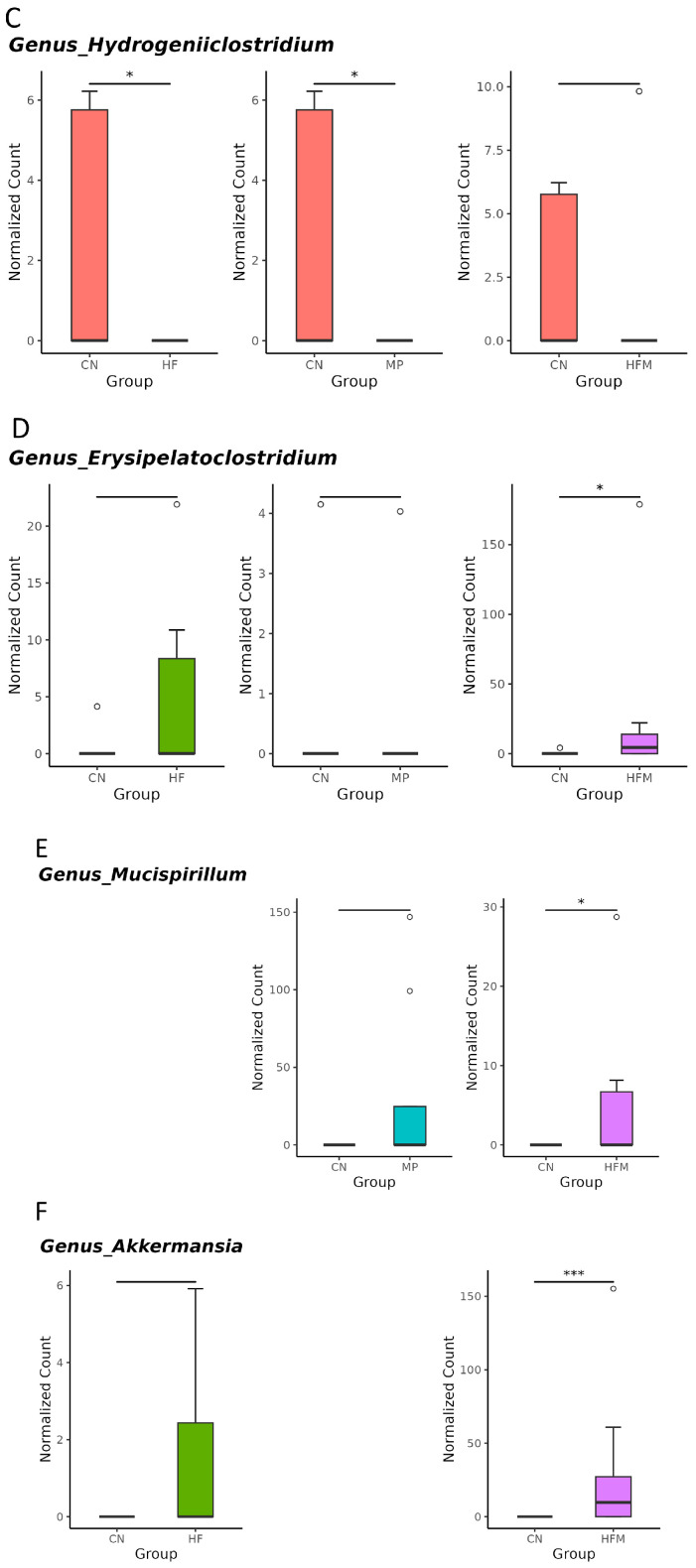
Genus-level taxa that significantly differed (false discovery rate (FDR) < 0.05) in the relative abundance of (**A**) *Odoribacter*, (**B**) *Fournierella*, (**C**) *Hydrogeniiclostridium*, (**D**) *Erysipelatoclostridium*, (**E**) *Mucispirillum*, and (**F**) *Akkermansia*. * *p* < 0.05. ** *p* < 0.01. *** *p* < 0.005. Outliers are denoted by dots. n = 7–8 per group.

Given that specific gut microbes participate in H_2_S metabolism [23] and BP regulation [24], we further examined the relative abundance of related genera. Although sulfate-reducing bacteria (SRB; e.g., *Desulfovibrio* spp.) were not significantly altered by maternal HF diet, H_2_S metabolism–associated genera such as *Odoribacter* (Figure 5A), *Fournierella* (Figure 5B), and *Hydrogeniiclostridium* (Figure 5C) were markedly depleted in the HF group compared with the control (CN) group. Similarly, MP exposure had minimal effects on SRB but reduced the abundance of *Fournierella* (Figure 5B) and *Hydrogeniiclostridium* (Figure 5C). Under combined HF+MP exposure, the pronounced decrease in *Fournierella* (Figure 5B) was accompanied by lower predicted H_2_S biosynthetic capacity, whereas the enrichment of *Erysipelatoclostridium* (Figure 5D), *Mucispirillum* (Figure 5E), and *Akkermansia* (Figure 5F) was associated with elevated BP [24].

### 3.4. Metabolomic Profile Analysis

In total, 2361 metabolites (1813 and 548 in positive and negative ionization mode, respectively) were detected using UHPLC-MS. These metabolites were further categorized into four confidence levels (59, 128, 797, and 1377 in levels 1–4, respectively). Volcano plot analysis was performed to identify significantly altered metabolites between each two group. PCA was performed to evaluate the metabolic differences between groups.

**Figure 6:**
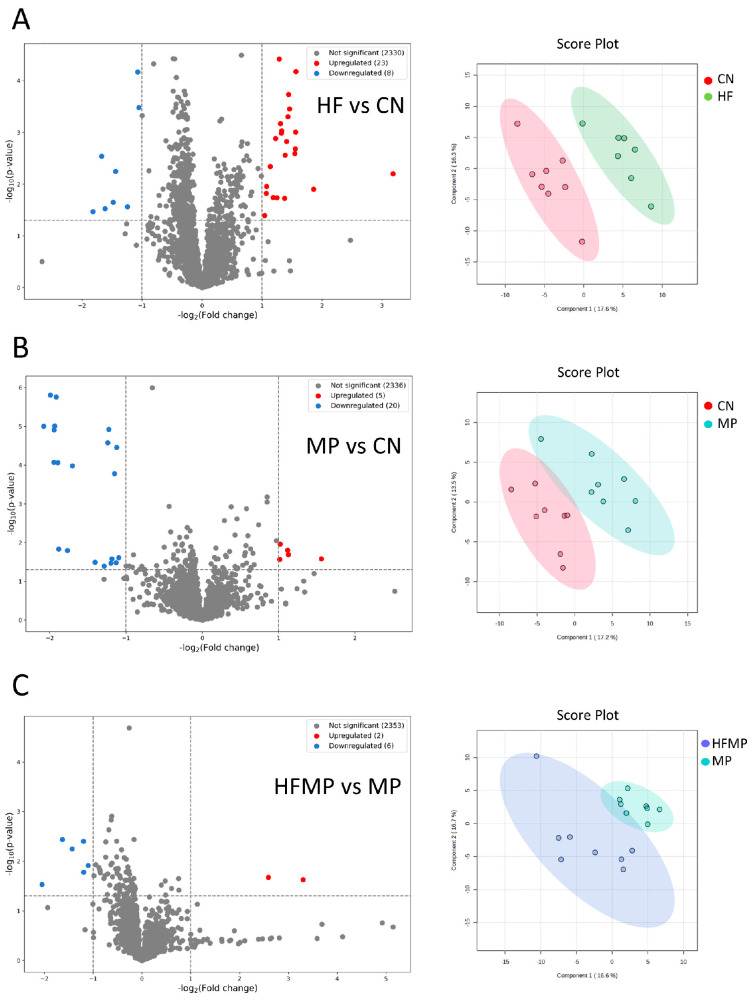
Volcano plots and principal component analysis (PCA) of offspring metabolic profiles. (**A**) HF versus control (CN). (**B**) MP versus CN. (**C**) HFMP versus MP. PCA plots demonstrate clear separation of metabolic profiles among the corresponding groups. n = 7–8 per group.

As shown in Figure 6A, comparison between the HF and CN groups identified 23 upregulated and eight downregulated metabolites. In the MP versus CN comparison, five metabolites were upregulated, and 20 were downregulated (Figure 6B). Additionally, the HFMP versus MP comparison revealed two upregulated and six downregulated metabolites (Figure 6C). PCA plots further demonstrated distinct separations in the metabolic profiles among the HF and CN groups (Figure 6A), the MP and CN groups (Figure 6B), and the HFMP and MP groups (Figure 6C).

**Figure 7:**
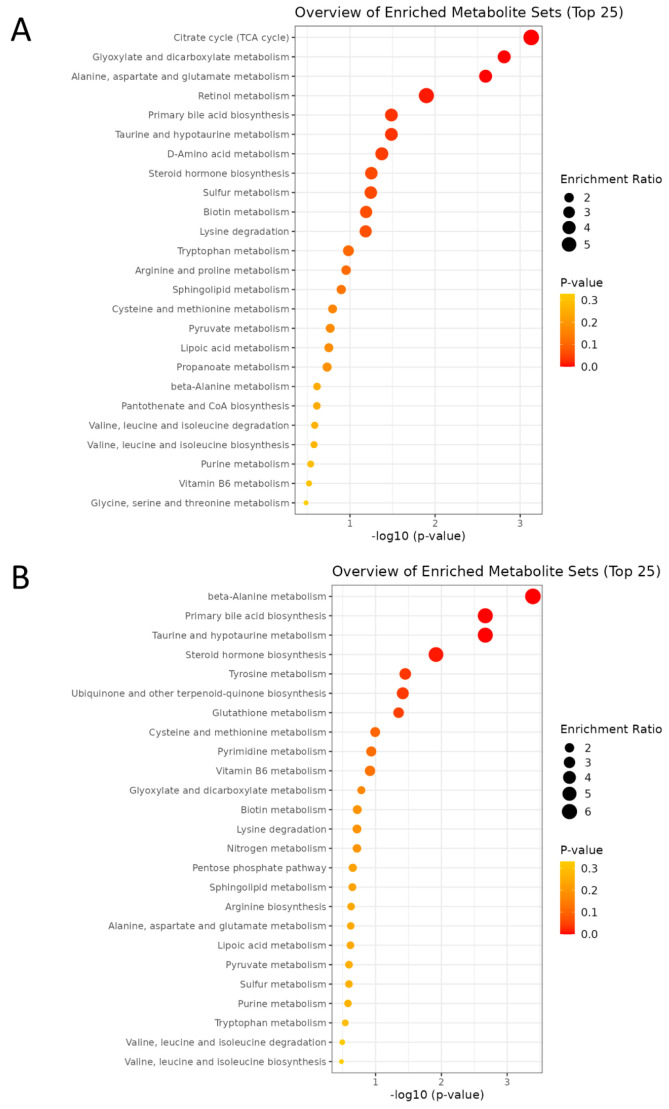
The enrichment analysis plot comparing the two groups was generated by MetaboAnalyst. (**A**) HF vs. CN. (**B**) MP vs. CN. n = 7–8 per group.

Enrichment analysis was performed to identify functionally related metabolites altered in our study. In pathway analysis, larger bubble size indicates greater impact, while deeper orange color represents a lower *p*-value. Maternal HF diet notably affected pathways including the TCA cycle, glyoxylate and dicarboxylate metabolism, alanine, aspartate, and glutamate metabolism, retinol metabolism, primary bile acid biosynthesis, and taurine/hypotaurine metabolism (Figure 7A), suggesting a coordinated disturbance in mitochondrial energy and redox homeostasis that links carbon–nitrogen–sulfur fluxes to cellular signaling and detoxification. In contrast, MP exposure significantly perturbed β-alanine metabolism, bile acid and taurine/hypotaurine metabolism, steroid hormone biosynthesis, tyrosine metabolism, ubiquinone and terpenoid-quinone biosynthesis, and glutathione metabolism (Figure 7B), reflecting mitochondrial dysfunction, oxidative stress, and dysregulated lipid–amino acid signaling.

## 4. Discussion

Although previous studies have reported that maternal HF diet or MP exposure independently induces adverse renal outcomes and hypertension in offspring [7,9], the present study is the first to demonstrate that combined HF and MP exposures during pregnancy and lactation synergistically exacerbate the risk of hypertension in adult offspring.

Key findings of this study are as follows: (1) adult offspring exhibited elevated blood pressure following maternal HF diet or MP exposure, with a significant interaction between the two exposures; (2) MP exposure increased glomerular and tubulointerstitial injury scores, indicating renal damage; (3) MP exposure suppressed renal H_2_S signaling, evidenced by reduced expression of H_2_S-generating enzymes and diminished in vitro H_2_S production; (4) maternal HF diet and MP exposure induced distinct gut microbiota profiles, altering taxa involved in H_2_S metabolism and BP regulation; (5) metabolomic profiling identified 2361 metabolites and demonstrated distinct metabolic alterations among the four groups, including those linked to H_2_S metabolism; and (6) pathway enrichment analysis revealed that both HF diet and MP exposure disrupted taurine/hypotaurine and sulfur-associated pathways, suggesting impaired H_2_S production and redox homeostasis.

Consistent with our previous findings, maternal MP exposure alone was sufficient to induce BP elevation and renal injury in adult offspring [10]. Similarly, maternal HF diet alone has been shown to increase offspring BP [16]. Within the framework of the DOHaD, our study provides new insights into how maternal HF diet and MP exposure synergistically induce adverse “kidney programming,” predisposing offspring to hypertension and renal injury [25]. The observed interaction in this “two-hit” model underscores an emerging environmental health concern, as many HFCS-containing beverages are packaged in plastics capable of leaching considerable amounts of MPs, thereby posing a pervasive exposure risk to women of reproductive age [5]. The MP dose used in our study (1 mg/L) was chosen to approximate environmentally relevant chronic exposures rather than acute toxicity. Environmental assessments of bottled waters have reported MP contamination on both particle and mass bases, including estimated concentrations of ~656 µg/L for MPs sized 0.5–10 µm following particle count-to-mass conversion [26] and lower microplastic levels in other beverages [27]. Although direct mass–particle comparisons remain challenging due to variability in particle size and polymer composition, these data indicate that MPs occur in drinking sources at tens to hundreds of micrograms per liter, supporting the translational relevance of our experimental exposure.

Although H_2_S signaling emerged as a unifying mechanism linking maternal insults to both offspring hypertension and kidney disease [15], its role in HF and MP exposures has not been explored yet. Our data was the first to indicate that maternal MP exposure affects the H_2_S pathways. Our study provides the first evidence that maternal MP exposure perturbs the renal H_2_S pathway. Specifically, MP exposure markedly downregulated the expression of the H_2_S-generating enzymes CBS and CSE, accompanied by a significant reduction in renal H_2_S production activity. Given the well-established antioxidant and BP–lowering properties of H_2_S, these findings suggest that maternal MP exposure-induced hypertension and renal injury in offspring may, at least in part, be mediated by dysregulation of the H_2_S system.

Alterations in the gut microbiota may contribute to kidney programming. Maternal HF diet and MP exposure, individually or in combination, reshaped the offspring gut microbiota, leading to distinct clustering patterns and exposure-specific microbial signatures. Several mechanisms may underlie these effects: HF intake can alter microbial composition by providing excess fructose that preferentially supports fructolytic bacteria and modulates short-chain fatty acid production, thereby influencing microbial metabolism and gut environment [28]. In contrast, MPs can serve as surfaces for bacterial attachment and biofilm formation, creating microhabitats that facilitate colonization by specific microbial taxa [29]. Additional mechanisms may include HF- or MP-induced alterations in gut redox state, local inflammation, or nutrient availability, which can further modulate microbial community structure [30,31]. Functional prediction analysis further revealed suppression of the sulfate assimilation and cysteine biosynthesis pathways, implicating impaired microbial H_2_S metabolism as a mechanistic link. Consistent with this notion, genera functionally associated with sulfur or cysteine metabolism—including *Odoribacter*, *Fournierella*, and *Hydrogeniiclostridium*—were depleted [32,33], particularly under combined HF+MP exposure, suggesting diminished microbial H_2_S-generating capacity. Conversely, enrichment of *Erysipelatoclostridium*, *Mucispirillum*, *Ruminococcus*, and *Akkermansia*—taxa previously linked to hypertension [24]—indicates a microbial signature that may contribute to elevated BP. Together, these findings support the concept that maternal HF and MP exposures converge to disrupt both host and microbial H_2_S-generating systems, thereby fostering an adverse gut–kidney environment predisposing offspring to hypertension and renal injury.

Untargeted metabolomic profiling revealed that maternal HF diet, MP exposure, or their combination induced distinct metabolic reprogramming in offspring. PCA showed clear group-specific clustering, reflecting pronounced perturbations in response to maternal nutritional and environmental insults. Maternal HF diet altered the TCA cycle, amino acid (alanine, aspartate, glutamate) metabolism, bile acid synthesis, and notably taurine/hypotaurine metabolism, while MP exposure affected taurine/hypotaurine and glutathione metabolism, β-alanine metabolism, steroid hormone biosynthesis, and redox-related pathways. Taurine/hypotaurine metabolism is directly linked to H_2_S synthesis via the transsulfuration pathway (CBS, CSE, 3-MST), whereas glutathione metabolism couples to H_2_S through persulfide formation (GSSH) and antioxidant regulation [34,35]. Perturbation of these sulfur-containing pathways, along with cysteine metabolism, suggests impaired H_2_S-generating capacity, consistent with reduced renal CBS/CSE expression and H_2_S production. Together, these findings suggest that maternal HF and MP exposures are associated with alterations in the H_2_S pathway, alongside metabolic dysregulation, oxidative stress, and changes in the gut microbiota that may relate to increased susceptibility to hypertension and kidney injury in offspring.

Several limitations of the present study should be acknowledged. First, although dysregulation of the H_2_S pathway provides a mechanistic link to maternal HF- and MP-induced hypertension, it may not fully account for the HF-mediated effects. Future studies exploring interactions between H_2_S signaling and other established mechanisms, such as oxidative stress and nitric oxide deficiency, could inform novel preventive and therapeutic strategies. A limitation of this study is that renal H_2_S alterations were inferred primarily from enzyme expression and activity rather than direct tissue measurements, and only canonical H_2_S-producing enzymes (CBS, CSE, 3MST) were assessed. Additional H_2_S-producing enzymes (CARS1/2) and detoxifying enzymes were not evaluated, which may limit a full understanding of sulfur-related redox homeostasis. Furthermore, our findings linking H_2_S signaling and gut microbiota to offspring hypertension are correlative, as no mechanistic interventions—such as H_2_S donor administration, microbial supplementation, or fecal microbiota transplantation—were performed. Future studies using derivatization- or gas-based H_2_S detection, comprehensive analysis of production and clearance pathways, and mechanistic interventions are warranted to clarify enzyme-specific contributions, redox balance, and the causal role of microbiota in hypertension. Additionally, assessing H_2_S in other BP-regulated organs, such as the vasculature and heart, in follow-up experiments will further strengthen the mechanistic link to BP regulation. Second, we did not assess gut microbiota or untargeted metabolomics in dams or neonatal offspring, limiting our ability to distinguish whether the observed changes in adult offspring reflect postnatal plasticity or programmed effects of maternal HF or MP exposure. Third, accurate quantification of MPs in maternal and offspring tissues remains technically challenging due to their small size, heterogeneity, and low abundance; direct measurements using techniques such as Raman spectroscopy were not performed [36]. Fourth, this study used sulfated microplastics, which may exert biological effects due to their sulfur groups, making it difficult to isolate the impact of the microplastic polymer itself. Future studies using non-sulfonated microplastics are needed to clarify this distinction. Finally, metabolomic alterations reflect integrated changes in gene and protein expression across multiple organs, yet our study focused primarily on the kidney, which may limit interpretation regarding other BP-regulating organ systems. Comprehensive multi-organ studies are warranted to further elucidate systemic effects.

## 5. Conclusions

In conclusion, combined maternal HF and MP exposures during pregnancy and lactation may increase risk of hypertension and kidney damage in adult offspring. These findings coincide with alterations in H_2_S signaling, gut microbiota composition, and metabolic profiles, suggesting that multiple interacting biological systems may be involved in developmental programming. Further investigation of these pathways may help inform future strategies to better understand, and potentially mitigate, the long-term kidney and cardiometabolic implications of early-life HF and MP exposure.

## Figures and Tables

**Table 1:** Effect of maternal high-fructose (HF) diet and microplastic (MP) exposure on weighs and blood pressure.

Group	CN	HF	MP	HFMP
N	8	7	8	8
Body weight (g)	463 ± 13	426 ± 17	447 ± 18	446 ± 16
Left kidney weight (KW) (g)	2.0 ± 0.04	1.85 ± 0.04	1.97 ± 0.07	1.97 ± 0.11
Left KW/100 g BW	0.43 ± 0.01	0.44 ± 0.01	0.44 ± 0.02	0.44 ± 0.02
Systolic BP (mmHg), week 3	103 ± 1	107 ± 1	108 ± 2	109 ± 1
Systolic BP (mmHg), week 4	104 ± 1	108 ± 1	108 ± 2	110 ± 1
Systolic BP (mmHg), week 8	119 ± 1	127 ± 1 *	127 ± 1 *	133 ± 1 *#$
Systolic BP (mmHg), week 12	130 ± 1	139 ± 1 *	143 ± 1 *	150 ± 2 *#$

* *p* < 0.05 vs. CN; # *p* < 0.05 vs. HF; $ *p* < 0.05 vs. MP.

## Data Availability

Data are contained within the article.

## References

[1] Lin Y.D., Huang P.H., Chen Y.W., Hsieh C.W., Tain Y.L., Lee B.H., Hou C.Y., Shih M.K. (2023). Sources, Degradation, Ingestion and Effects of Microplastics on Humans: A Review. Toxics.

[2] Lehner R., Weder C., Petri-Fink A., Rothen-Rutishauser B. (2019). Emergence of nanoplastic in the environment and possible impact on human health. Environ. Sci. Technol..

[3] Johnson R.J., Segal M.S., Sautin Y., Nakagawa T., Feig D.I., Kang D.H., Gersch M.S., Benner S., Sánchez-Lozada L.G. (2007). Potential role of sugar (fructose) in the epidemic of hypertension, obesity and the metabolic syndrome, diabetes, kidney disease, and cardiovascular disease. Am. J. Clin. Nutr..

[4] Sewwandi M., Wijesekara H., Rajapaksha A.U., Soysa S., Vithanage M. (2023). Microplastics and plastics-associated contaminants in food and beverages; Global trends, concentrations, and human exposure. Environ. Pollut..

[5] Popkin B.M., Hawkes C. (2016). Sweetening of the global diet, particularly beverages: Patterns, trends, and policy responses. Lancet Diabetes Endocrinol..

[6] Fleming T.P., Velazquez M.A., Eckert J.J. (2015). Embryos, DOHaD and David Barker. J. Dev. Orig. Health Dis..

[7] Jia G., Hill M.A., Sowers J.R. (2019). Maternal exposure to high fructose and offspring health. Hypertension.

[8] de Oliveira R.B., Pelepenko L.E., Masaro D.A., Lustosa G.M.M.M., de Oliveira M.C., Roza N.A.V., Marciano M.A., Dos Reis L.M., Kamel S., Louvet L. (2024). Effects of microplastics on the kidneys: A narrative review. Kidney Int..

[9] Moreno G.M., Brunson-Malone T., Adams S., Nguyen C., Seymore T.N., Cary C.M., Polunas M., Goedken M.J., Stapleton P.A. (2024). Identification of micro- and nanoplastic particles in postnatal sprague-dawley rat offspring after maternal inhalation exposure throughout gestation. Sci. Total Environ..

[10] Cheng Y.C., Chen W.L., Yu H.R., Tsai C.Y., Sheen J.M., Tiao M.M., Hsu C.N., Tain Y.L. (2025). Microplastic-induced hypertension in rats: A two-hit model exploring oxidative stress and gut microbiota. NanoImpact.

[11] Hou Y., Lv B., Du J., Ye M., Jin H., Yi Y., Huang Y. (2025). Sulfide regulation and catabolism in health and disease. Signal Transduct. Target Ther..

[12] Weber G.J., Pushpakumar S., Tyagi S.C., Sen U. (2016). Homocysteine and hydrogen sulfide in epigenetic, metabolic and microbiota related renovascular hypertension. Pharmacol. Res..

[13] Peleli M., Zampas P., Papapetropoulos A. (2022). Hydrogen Sulfide and the Kidney: Physiological Roles, Contribution to Pathophysiology, and Therapeutic Potential. Antioxid. Redox Signal..

[14] Tomasova L., Konopelski P., Ufnal M. (2016). Gut Bacteria and Hydrogen Sulfide: The New Old Players in Circulatory System Homeostasis. Molecules.

[15] Hsu C.N., Lin Y.J., Hou C.Y., Chen Y.W., Tain Y.L. (2025). Early-Life Hydrogen Sulfide Signaling as a Target for Cardiovascular-Kidney-Metabolic Syndrome Reprogramming. Antioxidants.

[16] Tain Y.L., Wu K.L., Lee W.C., Leu S., Chan J.Y. (2015). Maternal fructose-intake-induced renal programming in adult male offspring. J. Nutr. Biochem..

[17] An R., Wang X., Yang L., Zhang J., Wang N., Xu F., Hou Y., Zhang H., Zhang L. (2021). Polystyrene microplastics cause granulosa cells apoptosis and fibrosis in ovary through oxidative stress in rats. Toxicology.

[18] Olivera S., Graham D. (2023). Sex differences in preclinical models of hypertension. J. Hum. Hypertens..

[19] Douglas G.M., Maffei V.J., Zaneveld J.R., Yurgel S.N., Brown J.R., Taylor C.M., Huttenhower C., Langille M.G.I. (2020). PICRUSt2 for prediction of metagenome functions. Nat. Biotechnol..

[20] Hsu C.N., Lin Y.J., Lu P.C., Tain Y.L. (2018). Early Supplementation of d-Cysteine or l-Cysteine Prevents Hypertension and Kidney Damage in Spontaneously Hypertensive Rats Exposed to High-Salt Intake. Mol. Nutr. Food Res..

[21] Caspi R., Billington R., Keseler I.M., Kothari A., Krummenacker M., Midford P.E., Ong W.K., Paley S., Subhraveti P., Karp P.D. (2020). The MetaCyc database of metabolic pathways and enzymes—A 2019 update. Nucleic Acids Res..

[22] Parks D.H., Tyson G.W., Hugenholtz P., Beiko R.G. (2014). STAMP: Statistical analysis of taxonomic and functional profiles. Bioinformatics.

[23] Carbonero F., Benefiel A.C., Alizadeh-Ghamsari A.H., Gaskins H.R. (2012). Microbial pathways in colonic sulfur metabolism and links with health and disease. Front. Physiol..

[24] Palmu J., Lahti L., Niiranen T. (2021). Targeting Gut Microbiota to Treat Hypertension: A Systematic Review. Int. J. Environ. Res. Public Health.

[25] Tain Y.L. (2025). Advocacy for DOHaD research optimizing child kidney health. Pediatr. Neonatol..

[26] Zuccarello P., Ferrante M., Cristaldi A., Copat C., Grasso A., Sangregorio D., Fiore M., Oliveri Conti G. (2019). Exposure to microplastics (<10 μm) associated to plastic bottles mineral water consumption: The first quantitative study. Water Res..

[27] Kosuth M., Mason S.A., Wattenberg E.V. (2018). Anthropogenic contamination of tap water, beer, and sea salt. PLoS ONE.

[28] Westerbeke F.H.M., Rios-Morales M., Attaye I., Nieuwdorp M. (2025). Fructose catabolism and its metabolic effects: Exploring host-microbiota interactions and the impact of ethnicity. J. Physiol..

[29] Ventura E., Marín A., Gámez-Pérez J., Cabedo L. (2024). Recent advances in the relationships between biofilms and microplastics in natural environments. World J. Microbiol. Biotechnol..

[30] Thin Z.S., Chew J., Ong T.Y.Y., Raja Ali R.A., Gew L.T. (2025). Impact of microplastics on the human gut microbiome: A systematic review of microbial composition, diversity, and metabolic disruptions. BMC Gastroenterol..

[31] Hsu C.N., Yu H.R., Chan J.Y.H., Wu K.L.H., Lee W.C., Tain Y.L. (2022). The Impact of Gut Microbiome on Maternal Fructose Intake-Induced Developmental Programming of Adult Disease. Nutrients.

[32] Wallace J.L., Motta J.P., Buret A.G. (2018). Hydrogen sulfide: An agent of stability at the microbiome-mucosa interface. Am. J. Physiol. Gastrointest. Liver Physiol..

[33] Wolf P.G., Cowley E.S., Breister A., Matatov S., Lucio L., Polak P., Ridlon J.M., Gaskins H.R., Anantharaman K. (2022). Diversity and distribution of sulfur metabolic genes in the human gut microbiome and their association with colorectal cancer. Microbiome.

[34] Stipanuk M.H. (2020). Metabolism of Sulfur-Containing Amino Acids: How the Body Copes with Excess Methionine, Cysteine, and Sulfide. J. Nutr..

[35] Kimura H. (2015). Physiological Roles of Hydrogen Sulfide and Polysulfides. Chemistry, Biochemistry and Pharmacology of Hydrogen Sulfide; Handbook of Experimental Pharmacology.

[36] Barceló D., Picó Y., Alfarhan A.H. (2023). Microplastics: Detection in human samples, cell line studies, and health impacts. Environ. Toxicol. Pharmacol..

